# Localization of a Guanylyl Cyclase to Chemosensory Cilia Requires the Novel Ciliary MYND Domain Protein DAF-25

**DOI:** 10.1371/journal.pgen.1001199

**Published:** 2010-11-24

**Authors:** Victor L. Jensen, Nathan J. Bialas, Sharon L. Bishop-Hurley, Laurie L. Molday, Katarzyna Kida, Phuong Anh T. Nguyen, Oliver E. Blacque, Robert S. Molday, Michel R. Leroux, Donald L. Riddle

**Affiliations:** 1 Medical Genetics, University of British Columbia, Vancouver, Canada; 2 Molecular Biology and Biochemistry, Simon Fraser University, Burnaby, Canada; 3 Division of Biological Sciences, University of Missouri, Columbia, Missouri, United States of America; 4 CSIRO-Livestock Industries, Queensland Biosciences Precinct, Brisbane, Australia; 5 Centre for Macular Research, University of British Columbia, Vancouver, Canada; 6 School of Biomolecular and Biomedical Science, UCD Conway Institute, University College Dublin, Belfield, Dublin, Ireland; 7 Michael Smith Laboratories, University of British Columbia, Vancouver, Canada; University of California San Francisco, United States of America

## Abstract

In harsh conditions, *Caenorhabditis elegans* arrests development to enter a non-aging, resistant diapause state called the dauer larva. Olfactory sensation modulates the TGF-β and insulin signaling pathways to control this developmental decision. Four mutant alleles of *daf-25* (abnormal DAuer Formation) were isolated from screens for mutants exhibiting constitutive dauer formation and found to be defective in olfaction. The *daf-25* dauer phenotype is suppressed by *daf-10*/IFT122 mutations (which disrupt ciliogenesis), but not by *daf-6*/PTCHD3 mutations (which prevent environmental exposure of sensory cilia), implying that DAF-25 functions in the cilia themselves. *daf-25* encodes the *C. elegans* ortholog of mammalian Ankmy2, a MYND domain protein of unknown function. Disruption of DAF-25, which localizes to sensory cilia, produces no apparent cilia structure anomalies, as determined by light and electron microscopy. Hinting at its potential function, the dauer phenotype, epistatic order, and expression profile of *daf-25* are similar to *daf-11*, which encodes a cilium-localized guanylyl cyclase. Indeed, we demonstrate that DAF-25 is required for proper DAF-11 ciliary localization. Furthermore, the functional interaction is evolutionarily conserved, as mouse Ankmy2 interacts with guanylyl cyclase GC1 from ciliary photoreceptors. The interaction may be specific because *daf-25* mutants have normally-localized OSM-9/TRPV4, TAX-4/CNGA1, CHE-2/IFT80, CHE-11/IFT140, CHE-13/IFT57, BBS-8, OSM-5/IFT88, and XBX-1/D2LIC in the cilia. Intraflagellar transport (IFT) (required to build cilia) is not defective in *daf-25* mutants, although the ciliary localization of DAF-25 itself is influenced in *che-11* mutants, which are defective in retrograde IFT. In summary, we have discovered a novel ciliary protein that plays an important role in cGMP signaling by localizing a guanylyl cyclase to the sensory organelle.

## Author Summary

*C. elegans* mutants that either fail to form or arrest development as dauer larvae, a stress-resistant lifestage, usually have defects in genes involved in evolutionarily conserved signaling pathways. In this study, we identified the gene mutated in *daf-25* mutant strains, which inappropriately arrest as dauer larvae and are also defective in the sense of smell. The mammalian counterpart of DAF-25 is Ankmy2, a protein of unknown function that contains three ankyrin repeats and a zinc finger MYND domain, both of which are predicted to bind other protein(s). We show that DAF-25/Ankmy2 is required for the proper localization of a membrane-bound guanylyl cyclase—a class of protein that functions in cyclic GMP signaling—to cilia, which are conserved sensory organelles. We further demonstrate that mammalian Ankmy2 binds the retinal guanylyl cyclase GC1, suggesting a role for Ankmy2 in vision—which critically depends on cyclic GMP signal transduction—suggesting the potential involvement of Ankmy2 in human retinal disease, as well as other cilia-related diseases such as obesity.

## Introduction

The dauer larva of *Caenorhabditis elegans* is an alternate third larval stage where a stress resistant, non-aging life plan is adopted in harsh environmental conditions [1]. Dauer larvae disperse and will resume development when conditions improve. The study of dauer formation has elucidated a complex gene network used to control the decision to go into diapause [2]. The dauer pathway includes well-recognized members in the canonical TGF-β (Transforming Growth Factor-Beta) and Insulin/Insulin-like signaling (IIS) pathways, as well as proteins affecting olfactory reception, neuron depolarization and peptide hormone secretion. Many mutants isolated as dauer formation defective (Daf-d) or constitutive (Daf-c) have revealed the key signaling components [2]. Here we identify DAF-25, a novel member of the olfactory signaling pathway that is associated with cGMP signaling—a signal transduction pathway with established links to cilia [3]. We show that the mammalian ortholog, Ankmy2, is expressed in ciliary photoreceptors and interacts with a guanylate cyclase (GC1), as predicted from the *C. elegans* results.

The olfactory signaling cascade has been well characterized in the two *C. elegans* amphids, organs consisting of a set of twelve bilaterally symmetric pairs of ciliated sensory neurons [4], [5]. While similar to mammalian olfactory signaling, at least some proteins involved are also homologous to those implicated in mammalian phototransduction [6]. Chemicals are sensed at the afferent, ciliated ends of sensory neurons where they contact the environment through pores in the cuticle. The cilia are required for chemosensation of chemical attractants and repellants, as well as for dauer entry and exit [7]. For many odorants the specific neurons that detect the odor are known [4]. For example, the AWA, AWB and AWC neuron pairs sense volatile odorants such as pyrazine, benzaldehyde, trimethyl thiazole and isoamyl alcohol. The ASH pair of ciliated olfactory neurons can detect changes in osmotic pressure.

The connection between dauer formation, chemosensory behavior and cilia is well known [2], [8]. *C. elegans* hermaphrodites only possess non-motile (primary) cilia which are found at the dendritic ends of 60 sensory neurons in the head and tail [5], [8]. Intraflagellar transport (IFT) proteins, normally required for building cilia, are well conserved in *C. elegans* and several have been discovered in this organism through the identification of sensory mutants [9]. Indeed, dauer formation is a sensory behavior dependent on the balanced inputs of dauer pheromone, temperature and food signals [4].

Proteins in the olfactory component of the dauer pathway include SRBC-64 and SRBC-66 (dauer pheromone receptors), DAF-11, a guanylyl cyclase, G-proteins (*gpa-2* and *gpa-3*), the Hsp90 molecular chaperone DAF-21, the IFT protein DAF-10, and the DAF-19 RFX-type transcription factor [10]–[14]. DAF-19 is strictly required for cilium formation as it regulates the expression of many cilia-related genes through a consensus sequence dubbed ‘x-box’ [15]. *daf-11*, *daf-19* and *daf-21* are Daf-c, whereas *daf-6* and *daf-10* are Daf-d [16]. *daf-19*, *daf-6* and *daf-10* are all dye-filling defective, indicating that their cilia (if present) are not exposed to the environment [17], [10]. By contrast, *daf-11* and *daf-21* mutants show wild-type dye filling [18]. All five mentioned *daf* genes are defective in recovery from the dauer diapause, presumably because they cannot detect the bacterial food stimulus [17]. Dauer recovery defects are present for mutants with broad chemosensory defects caused by abnormal ciliogenesis or signaling, and for many Unc genes, such as *unc-31*, which encodes a dense core vesicle secretion protein [17], [19], [20]. Our genetic screen for *C. elegans* Daf mutants has uncovered a novel ciliary protein, DAF-25, which participates in cGMP-associated signaling by modulating the ciliary localization of a guanylyl cyclase, DAF-11. The mammalian ortholog of DAF-25, Ankmy2, interacts with ciliary photoreceptor guanylyl cyclase 1 (GC1), indicating that the role of the MYND domain protein in cilia function is likely to be conserved and potentially relevant to human retinal disease or other ciliopathies.

## Results

### Genetic Epistasis Analysis Places DAF-25 Function in the Amphid Cilia

To identify genes potentially implicated in sensory transduction, we uncovered four alleles of *daf-25* in various screens for new mutants exhibiting a temperature-sensitive Daf-c phenotype. Three alleles (*m98*, *m137*, and *m362*) were isolated from ethyl methanesulfonate (EMS) mutagenesis screens and the fourth, *m488*, was isolated in a screen for Daf-c mutants with transposon insertions [21], [22].

Epistasis tests with the Daf-d mutants *daf-12*, *daf-16*, *daf-3*, *daf-6* and *daf-10* were used to position *daf-25* into the existing genetic pathway. Mutations in the *daf-12* nuclear hormone receptor gene suppress most Daf-c mutants [16], [23] including *daf-25* (0% dauer larva formation, n>200 for *daf-25(m362)*; *daf-12(m20)* compared to 97.5%, n = 281 for *daf-25(m362)* at 25°C). DAF-16/FOXO is the major downstream effector for Insulin/IGF1 signaling [24] as is DAF-3/Co-Smad for the TGF-β pathway [25]. Mutations in *daf-16* and *daf-3* only partially suppress the Daf-c phenotype of *daf-25* (37.6% dauer larvae, n = 407 for *daf-25(m362)*; *daf-16(m26)* and 60.0%, n = 167 for *daf-25(m362)*; *daf-3(mgDf90)* at 25°C), indicating that DAF-25 likely functions upstream of both pathways.

Importantly, *daf-10*, which encodes an IFT protein (DAF-10/IFT122) required for ciliogenesis [11], suppresses *daf-25* (0% dauer larvae, n>200 for *daf-25(m362)*; *daf-6(e1387)* compared to 97.5%, n = 281 for *daf-25(m362)* at 25°C), suggesting a function for DAF-25 within sensory cilia. *daf-6* mutants have closed amphid channels and cannot smell chemoattractants or form dauer larvae even though their cilia are present [5]. Interestingly, *daf-6* mutations do not suppress the *daf-25* Daf-c phenotype (97.4% dauer larvae, n = 312 for *daf-25(m362)*; *daf-6(e1377)* at 25°C), indicating that DAF-25 acts downstream of DAF-6, and that environmental (ciliary) input is not required for the Daf-c phenotype. DAF-6/PTCHD3 is expressed in the glial (sheath) cell that forms the amphid sensory channel, allowing contact of the sensory cilia to the environment through pores in the cuticle [26]. 8-bromo-cGMP rescues the dauer phenotype of *daf-25* (0% dauer larva formation for *daf-25(m362)* on 8-bromo-cGMP, n = 72 compared to 32% dauer larva formation on the control, n = 65, both at 20°C), similar to that previously reported for *daf-11* [12] indicating that DAF-25 functions upstream of the cGMP pathway in the cilia. Indeed the Daf-c phenotype of *daf-25(m362)* is very similar to that of *daf-11(m84)* at all temperatures tested (Table S1). The epistasis results are also similar to those for *daf-11*, indicating that both genes function at the same point in the genetic pathway—upstream of cilia formation and cGMP signaling in the cilia, and downstream of environmental input.

### *daf-25* Mutants Exhibit Chemosensory Phenotypes Independent of Ciliary Ultrastructure Defects

*daf-25* mutants are temperature-sensitive Daf-c and defective in dauer recovery. They constitutively form virtually 100% dauer larvae at 25°C, which do not recover upon transfer to 15°C. The Daf-c phenotype is rescued by maternally contributed *daf-25* as seen in the progeny of *daf-25(m362)* heterozygous hermaphrodites which form zero percent dauer larvae at 25°C (n>200). Moreover, *daf-25* animals exhibit defective responses to various chemosensory stimuli as well as a moderate defect in response to osmotic stress (37 of 45 *daf-25(m362)* adults crossed the sucrose hyperosmotic boundary compared to 1 of 45 for N2, χ^2^-p-value  = <0.00001, while 0 of 30 *daf-25(m362)* and N2 adults crossed a glycerol boundary). Adults are also defective in egg laying. Despite the fact that Daf-c genes in the IIS pathway (like *daf-2* and *age-1*) extend adult lifespan [27], *daf-25* mutants show no significant difference in lifespan from N2 (Figure S1).

**Figure 1:**
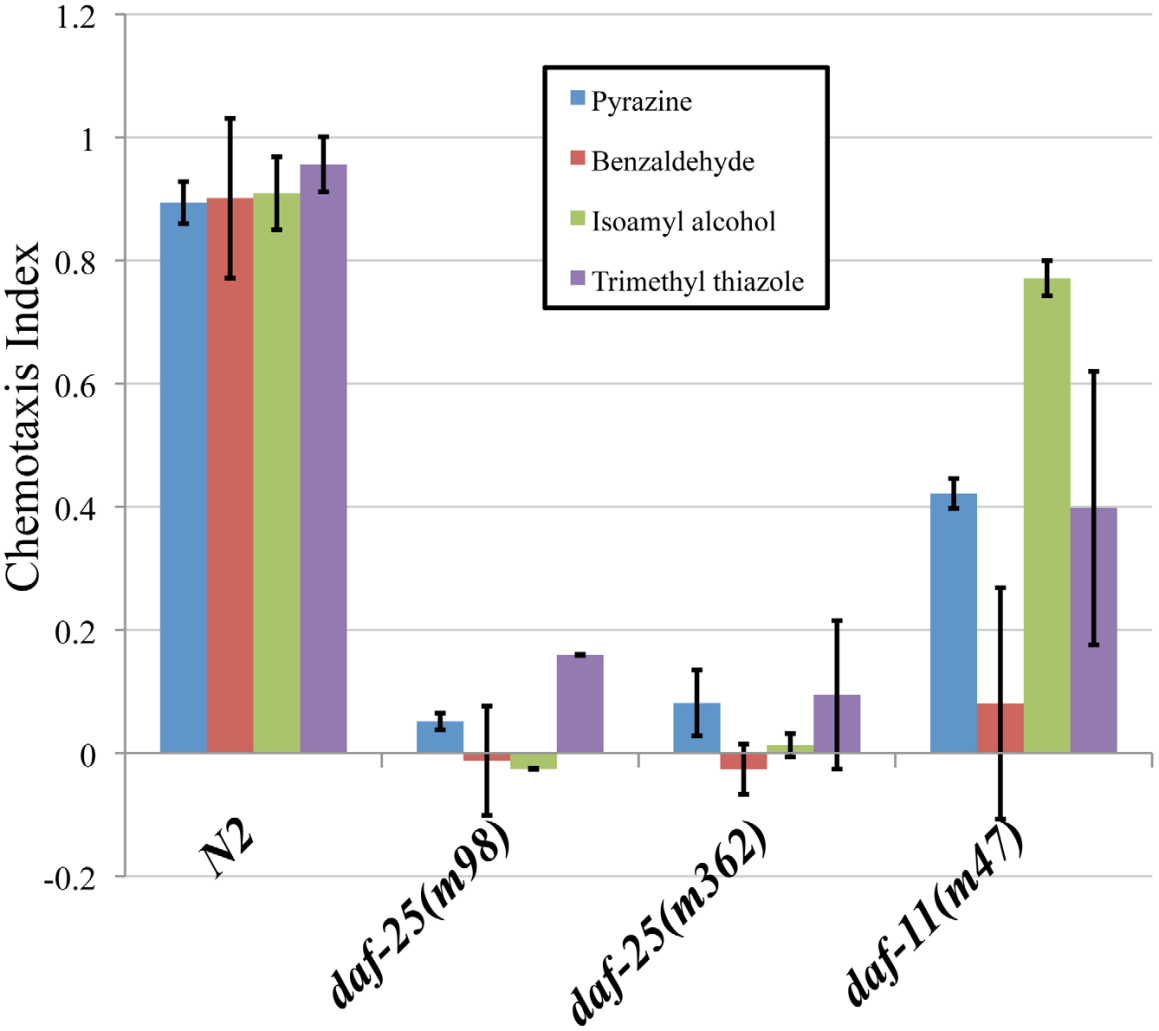
*daf-25* mutants have chemosensory defects. We assayed the ability of two *daf-25* mutants to respond to four attractants. The *daf-25* behavior was compared with N2 and with *daf-11(m47)*, which is partially chemotaxis defective. Chemotaxis index scores were calculated as the number of adults at the attractant minus the number at the control, divided by the total number of adults [50]. Neither allele of *daf-25* responded to any of the attractants, indicating an olfactory defect in *daf-25* mutants that is more severe than that of the *daf-11* guanylyl cyclase mutant. Benzaldehyde, trimethyl thiazole and isoamyl alcohol are detected by the AWC neurons, and pyrazine by the AWA neurons. Pyrazine: N2 n = 66, *daf-25(m98)* n = 78, *daf-25(m362)* n = 123, *daf-11(m47)* n = 83. Benzaldehyde: N2 n = 91, *daf-25(m98)* n = 80, *daf-25(m362)* n = 115, *daf-11(m47)* n = 62. Isoamyl alchohol: N2 n = 66, *daf-25(m98)* n = 78, *daf-25(m362)* n = 78, *daf-11(m47)* n = 83. Trimethyl thiazole: N2 n = 91, *daf-25(m98)* n = 69, *daf-25(m362)* n = 74, *daf-11(m47)* n = 93.

*daf-25* mutants are defective in chemotaxis to at least four volatile odorants (Figure 1). Wild-type N2 adults were attracted to the compounds tested, the chemotaxis-defective mutant *daf-11* was partially attracted, whereas the two *daf-25* mutants tested were nearly unresponsive (Figure 1). DAF-11 and the cGMP pathway are known to regulate responses to the AWC neuron-mediated odors isoamyl alcohol, trimethyl thiazole and benzaldehyde, and our results indicate that DAF-25 is also required in this pathway [28]. The AWA-detected scent, pyrazine, is not reported to be detected by the cGMP pathway, suggesting that DAF-25 participates in another signaling pathway in AWA neurons. Interestingly, alhough it has been shown that the cGMP pathway does not participate in AWA-mediated olfaction, the particular tested allele *daf-11(m47)* was previously shown to have reduced affinity for pyrazine [28], as we have seen here.

To establish if the olfactory phenotypes are associated with ciliary defects, mixed-stage populations of *daf-25* mutants and N2 were stained with the lipophillic dye, DiI. Mutants with cilia structure anomalies have abrogated dye filling of the olfactory neurons [29], whereas *daf-25* mutants take up the dye normally at all ages, suggesting that they have structurally intact cilia (Figure S2). To confirm this possibility, we further examined the integrity of ciliary structures by transmission electron microscopy. Ciliary ultrastructures in two *daf-25(m362)* L2 larvae—including transition zones, middle segments consisting of doublet microtubules, and distal segments composed of singlet microtubules—was indistinguishable from the two N2 controls (Figure S3). We conclude that *daf-25* animals have no obvious defects in ciliogenesis or cilia ultrastructure.

### Molecular Identification of *daf-25*

**Figure 2:**
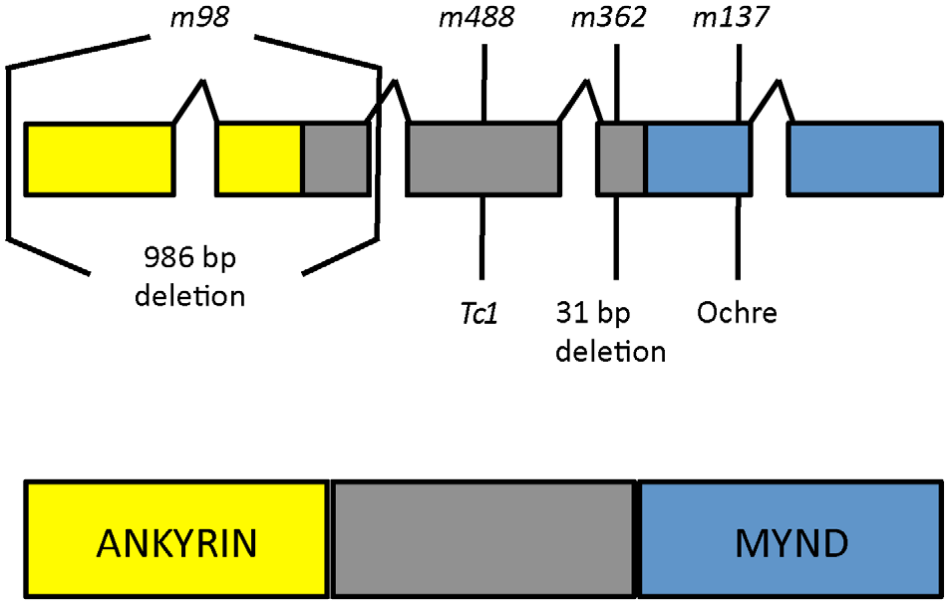
*daf-25* alleles encode the ortholog of mammalian Ankmy2. We identified the *daf-25* gene using three-factor genetic crosses and SNP mapping followed by ArrayCGH. The four alleles of *daf-25* include two EMS-induced deletions *m98* (996 bp deletion at I:332481-333477) and *m362* (31 bp deletion at I:335814-335844 which results in a premature stop 14 codons downstream), a transposon (Tc1) insertion at I:330927 (*m488*) and an EMS-induced ochre nonsense mutation *m137* (at I:336013). DAF-25 is well conserved and has been named Ankmy2 in mammals for its three ankyrin repeats and MYND-type zinc finger domain.

To identify the *daf-25* genetic locus, we first used three-factor genetic crosses to map the *m362* allele to the left arm of Chromosome I. Then, we employed a modified SNP mapping procedure [30], in which we selected for recombinants in the *unc-11-daf-25* interval to map *daf-25* to the left-most 1 Mbp of Chromosome I. Finally, we used a custom-made high-density array for the left-most ∼2.5 Mbp for comparative genomic hybridization (CGH). Two molecular lesions in *daf-25(m362)* were identified in exon 4 of Y48G1A.3 (Figure 2), including a 31 bp deletion and a G>A change 72 bp to the right of the deletion. Subsequent sequencing of PCR products from mutant genomic DNA uncovered the lesions in the remaining alleles. The *m98* mutant has a 996 bp deletion that removes the first two exons, *m137* has an ochre stop in the fourth exon, and *m488* has a Tc1 transposon insertion in the third exon (Figure 2).

Y48G1A.3 encodes the *C. elegans* ortholog of mammalian Ankmy2 (by reciprocal BLAST), a protein with three ankyrin repeats and a MYND-type zinc finger domain. The *C. elegans* protein shares throughout its length (388 residues) 52% similarity and 32% identity with mouse Ankmy2 (440 residues). The *C. elegans* ankyrin repeat domain is 40% identical and the MYND domain is 55% identical to the murine ortholog. Ankmy2 is very well conserved among chordates, with identity percentages compared to human Ankmy2 of 99% for macaque, 93% for cow, 88% for mouse, and 76% for zebrafish (Figure S4). Although the protein is highly conserved, there is no reported functional data for this gene from any organism. The MYND domain is thought to function in protein-protein interactions, although only a small number of MYND domain-containing proteins have been characterized, including the AML1/ETO protein, which binds SMRT/N-CoR through its MYND domain [31].

To analyze the transcript(s) generated by the *daf-25* gene, we employed a PCR-based approach. Using primers for the SL1 transplice sequence or poly-T in combination with gene-specific primers, we were able to amplify only one isoform (Figure S5). This result is consistent with the RNA-Seq and trans-splice data found on Wormbase, which shows a *daf-25* transcript sequence identical to that presented in Figure S5, including the 5′ and 3′ UTRs [32]–[34]. We were unable to amplify an SL2 trans-spliced product using multiple gene specific primers and an SL2 primer under any conditions tested.

### DAF-25 Functions in Cilia

**Figure 3:**
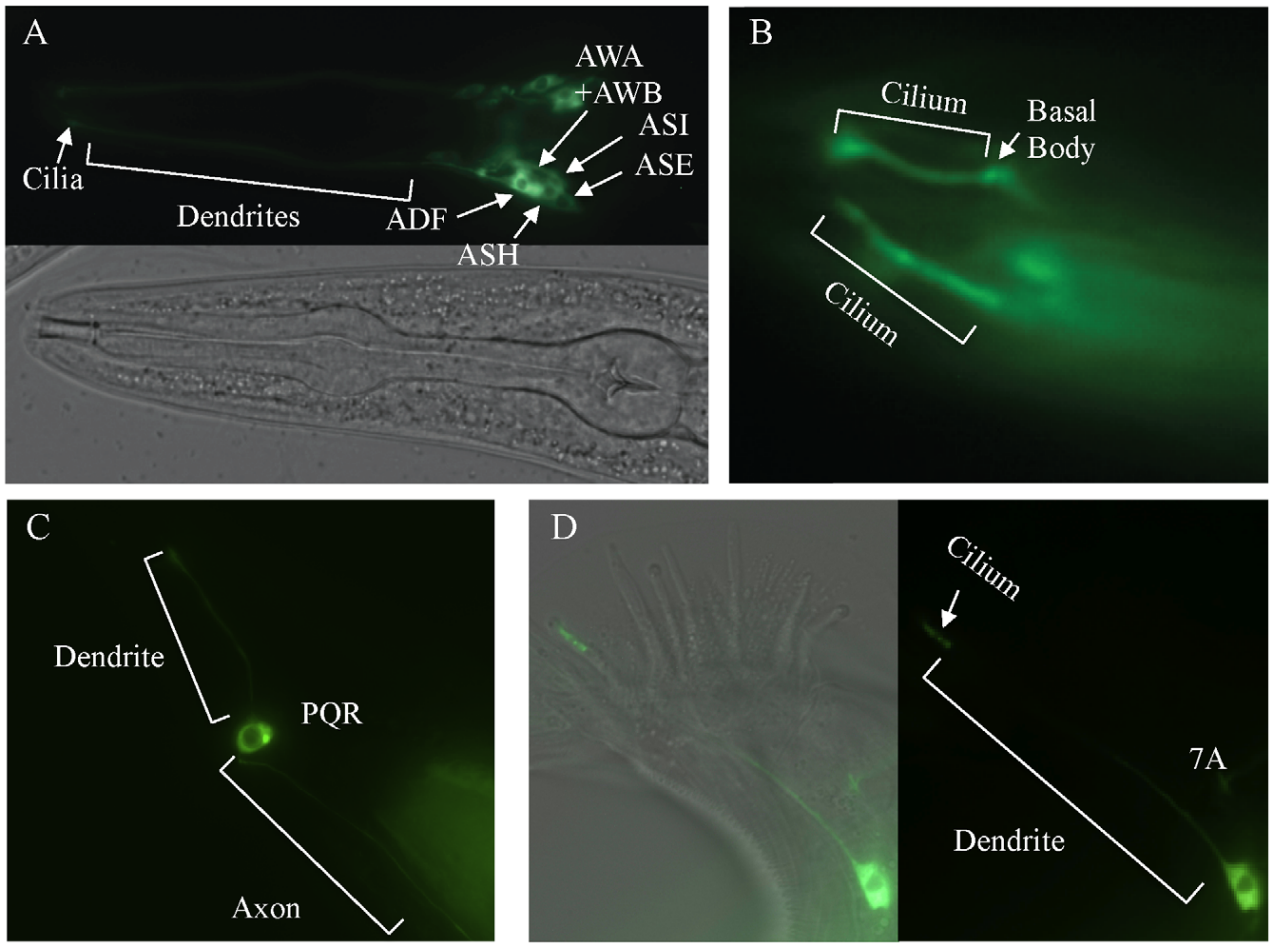
*daf-25* is expressed in many ciliated cells and encodes a novel ciliary protein. A reporter construct joined the 2.0 kb promoter region 5′ of the AUG for *daf-25* to the *daf-25* cDNA with the C-terminal GFP coding sequence. Expression is seen in many anterior chemosensory neurons in (A) including AFD, ASK, ASI, ASH, ASJ, ASG, ASE, ADF, AWA, AWB, AWC and IL2. There is a strong DAF-25::GFP signal localized in the sensory cilia (B). Expression of DAF-25::GFP is shown in the PQR neuron (C) and in the male tale neuron 7A (D).

To determine the sub-cellular localization of DAF-25, a GFP-tagged protein was constructed. The *daf-25* upstream promoter (approx. 2.0 kb 5′ of the ATG) was fused to the *daf-25* cDNA in frame into the pPD95.77 vector (gift from Dr. Andrew Fire) containing GFP (without a nuclear localization signal) and the *unc-54* 3′UTR. This construct was found to be expressed in many ciliated sensory neurons, including the following pairs of anterior neurons: AFD, ASK, ASI, ASH, ASJ, ASG, ASE, ADF, AWA, AWB, AWC and IL2 (Figure 3). It is also expressed in the PQR ciliated neuron and one ventral interneuron. We also show expression of the DAF-25::GFP construct in the 7A ciliated neuron in the male tail though we did not fully examine male expression due to the limited number examined and the mosaic expression associated with extra-chromosomal arrays. Most importantly, the fluorescence of the GFP-tagged protein was localized to the cilia of all these cells. The GFP-fusion construct was judged to be functional because it fully rescued the Daf-c phenotype of *daf-25(m362)* at 25°C while non-transgenic siblings arrested as dauers (n>200).

**Figure 4:**
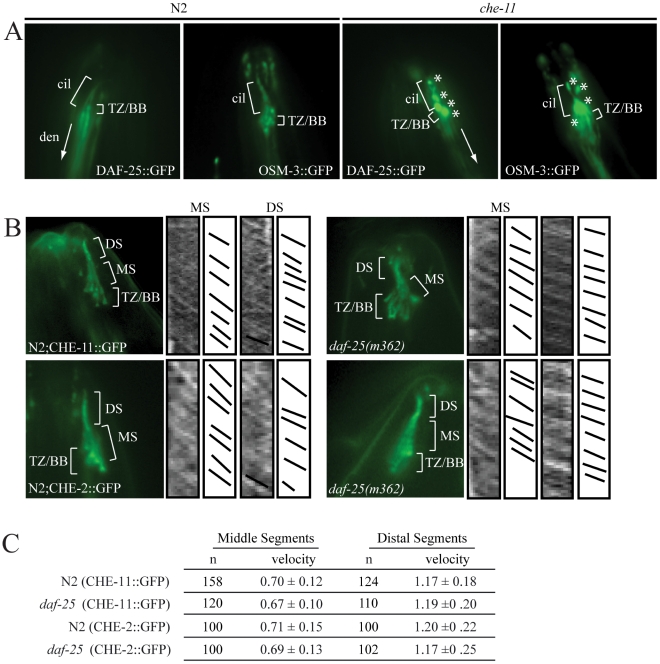
DAF-25 depends on IFT for proper localization within cilia but is not essential for the IFT process. The DAF-25::GFP translational fusion was crossed into *che-11(e1810)* and assayed for protein accumulation. As seen in (A), DAF-25::GFP localized normally to the cilia in the N2 background, but in the *che-11* background DAF-25::GFP accumulates in the cilia, indicating that when IFT is disrupted DAF-25 localization is also disrupted. We conclude that DAF-25 requires the IFT complex for proper transport and/or localization within cilia. Translational fusion reporters for CHE-2::GFP and CHE-11::GFP were crossed into *daf-25(m362)*. As reported previously [34] both reporters localize to basal bodies and ciliary axonemes (B), and have normal velocities in both N2 and *daf-25* mutants, as measured in the kymographs (C). Slopes in kymographs correlate with IFT complex speeds and were created as described previously [49]. In *daf-25(m362)* mutants there is no change in localization (B) or velocity (C) for either of the two reporters, indicating that DAF-25 is not required for normal rates of IFT transport, and is probably not a core IFT protein. cil  =  cilia, den  =  dendrite, TZ/BB  =  transition zone/basal body, asterisk indicates DAF-25::GFP accumulation, DS  =  distal segment, and MS  =  middle segment.

To investigate whether the ciliary localization of DAF-25 might depend on the intraflagellar transport (IFT) machinery, the DAF-25::GFP construct was crossed into *che-11*, which is required for retrograde transport in the cilia. In *che-11* mutants, IFT-associated proteins accumulate in the cilia [35]. The DAF-25::GFP translational fusion protein accumulated within the cilia and basal body (base of cilia) despite a reduction in total GFP fluorescence (mean DAF-25::GFP fluorescence in *che-11* (8.7E12) compared to N2 (1.4E13), p<0.00001, n = 9 for both), suggesting that the protein is associated with IFT (Figure 4). To test for a possible role for DAF-25 in the core IFT complex, GFP translational fusion constructs of two IFT proteins, CHE-2 and CHE-11 [30], were crossed into the *daf-25(m362)* mutant background and analyzed by time-lapse microscopy. The velocities of IFT transport of CHE-2 and CHE-11, as determined by kymograph analysis, were unchanged in *daf-25* compared to that of wild type animals (Figure 4). Specifically, transport velocities in the middle segment were ∼0.7 µm/s, and in the distal segments ∼1.2 µm/s, exactly as reported for all studied IFT proteins [36]. Collectively, our data show that DAF-25 is not essential for IFT, and is therefore unlikely to be a core component of IFT transport particles—consistent with the findings that the ciliary ultrastructure of the *daf-25* mutant is intact (Figure S3). However, its accumulation within cilia in the retrograde IFT mutant does suggest that it is associated with (i.e., transported by) the IFT machinery.

### DAF-25 Is Required for DAF-11 Localization to Cilia

**Figure 5:**
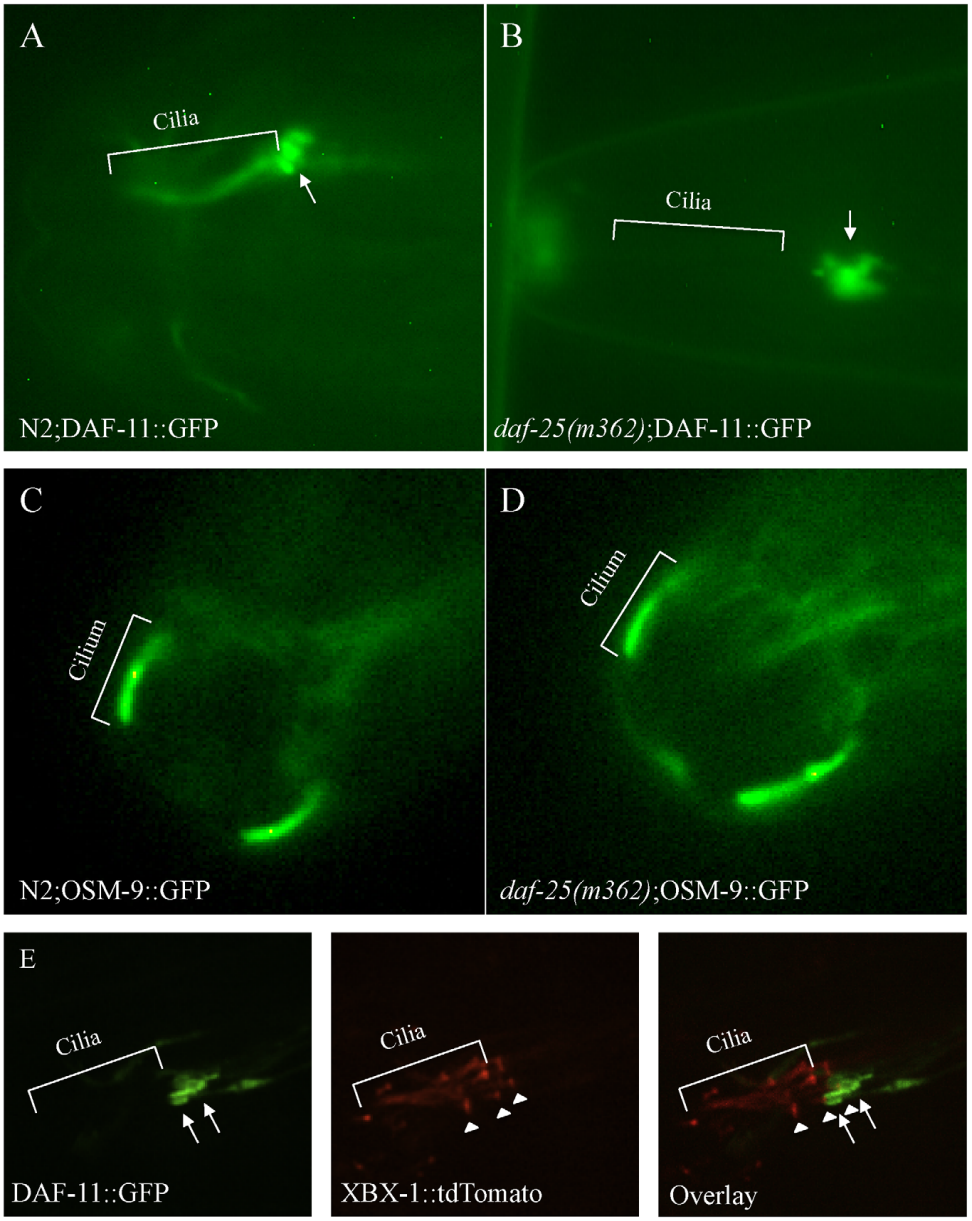
DAF-25 is required for the localization of a guanylyl cyclase (DAF-11) to cilia. The guanylyl cyclase DAF-11::GFP translational fusion protein was expressed in both N2 and *daf-25(m362)* genetic backgrounds. In wild type (A), DAF-11::GFP was localized to the ASI, ASJ or ASK sensory cilia, but was limited to the distal end of the dendrites (indicated by arrow) and largely excluded from basal body-ciliary structures in *daf-25(m362)* cilia (B). Normal ciliary localization was seen for the transient receptor potential channel (TRPV4) OSM-9::GFP reporter gene in both wild type (C) and *daf-25(m362)* (D). Also, no change in localization was seen for TAX-4/CNGA1, CHE-2/IFT80, CHE-11/IFT140, CHE-13/IFT57, BBS-8/TTC8, OSM-5/IFT88 and XBX-1/D2LIC in *daf-25* mutants (Figure S6). In (E), DAF-11::GFP and XBX-1::tdTomato are co-expressed in the amphid cilia in *daf-25(m362)* mutants. XBX-1::tdTomato localizes to the basal body (indicated by arrowhead) and cilia while DAF-11::GFP localizes to the distal end of the dendrite (indicated by arrow). No overlap in protein localization is observed indicating that DAF-11::GFP shows very little, if any localization to the basal body and no expression in the cilia. XBX-1::tdTomato is expressed in all of the amphid cilia while DAF-11::GFP is expressed in a subset.

The phenotype of *daf-25* is most similar to that of *daf-11*, and our epistasis results placed *daf-25* at the same position in the genetic pathway previously reported for *daf-11* [37]. To test for possible functional interactions, a strain harboring DAF-11::GFP (gift from Dr. James Thomas), which is known to localize to cilia [12], was crossed with two *daf-25* mutants (*m98* and *m362*). In wild-type animals, the DAF-11::GFP protein localized to the sensory cilia of the olfactory neuron pairs ASI, ASJ, ASK, AWB and AWC (Figure 5A), all of which express DAF-25::GFP (Figure 3). In both *daf-25* mutants, the DAF-11::GFP protein was observed only in a region near the base of cilia, rather than along their length (Figure 5B). To assess more precisely where the DAF-11::GFP protein is mislocalized, we introduced into the same strain a ciliary (IFT) marker, namely tdTomato-tagged XBX-1 (a gift from Dr. B. Yoder), which localizes at basal bodies and along the ciliary axoneme [38]. Visualization of the two fluorescently-tagged proteins in the *daf-25* mutant revealed that DAF-11::GFP accumulates at the very distal end of dendrites, with little or no localization to the basal body-ciliary structures (Figure 5E). This indicates that DAF-25 is required for the proper localization of DAF-11 to the cilia, providing a likely explanation for the similarities between the *daf-11* and *daf-25* mutant phenotypes. To test if the DAF-25-DAF-11 functional interaction is specific, GFP-tagged ciliary channel proteins (OSM-9/TRPV4 and TAX-4/CNGA1) and IFT-associated proteins (CHE-2/IFT80, CHE-11/IFT140, CHE-13/IFT57, BBS-8/TTC8, OSM-5/IFT88 and XBX-1/D2LIC) were also crossed into the *daf-25(m362)* mutant background. All eight reporters showed normal localization to the olfactory cilia in the wild-type N2 and *daf-25(m362)* strains, indicating the possible specificity of DAF-25 for guanylyl cyclases (OSM-9::GFP localization in the *daf-25* mutant shown in Figure 5C and 5D; the remaining constructs are presented in Figure S6). The mislocalization of DAF-11::GFP in *daf-25(m362)* was not suppressed by *daf-12(sa204)* (Figure S7). This indicates that it is the abrogation of DAF-25 rather than entry into dauer that controls the ciliary localization of DAF-11.

The GFP reporter results suggest a potentially specific function for DAF-25 in cilia. This finding is consistent with the reported regulation of *daf-25* by the ciliogenic DAF-19 RFX-type transcription factor [39]. Taken together, DAF-25 appears to be an adaptor protein required for the transport or tethering of the guanylyl cyclase DAF-11 within sensory cilia.

### Conservation of Function for DAF-25/Ankmy2

**Figure 6:**
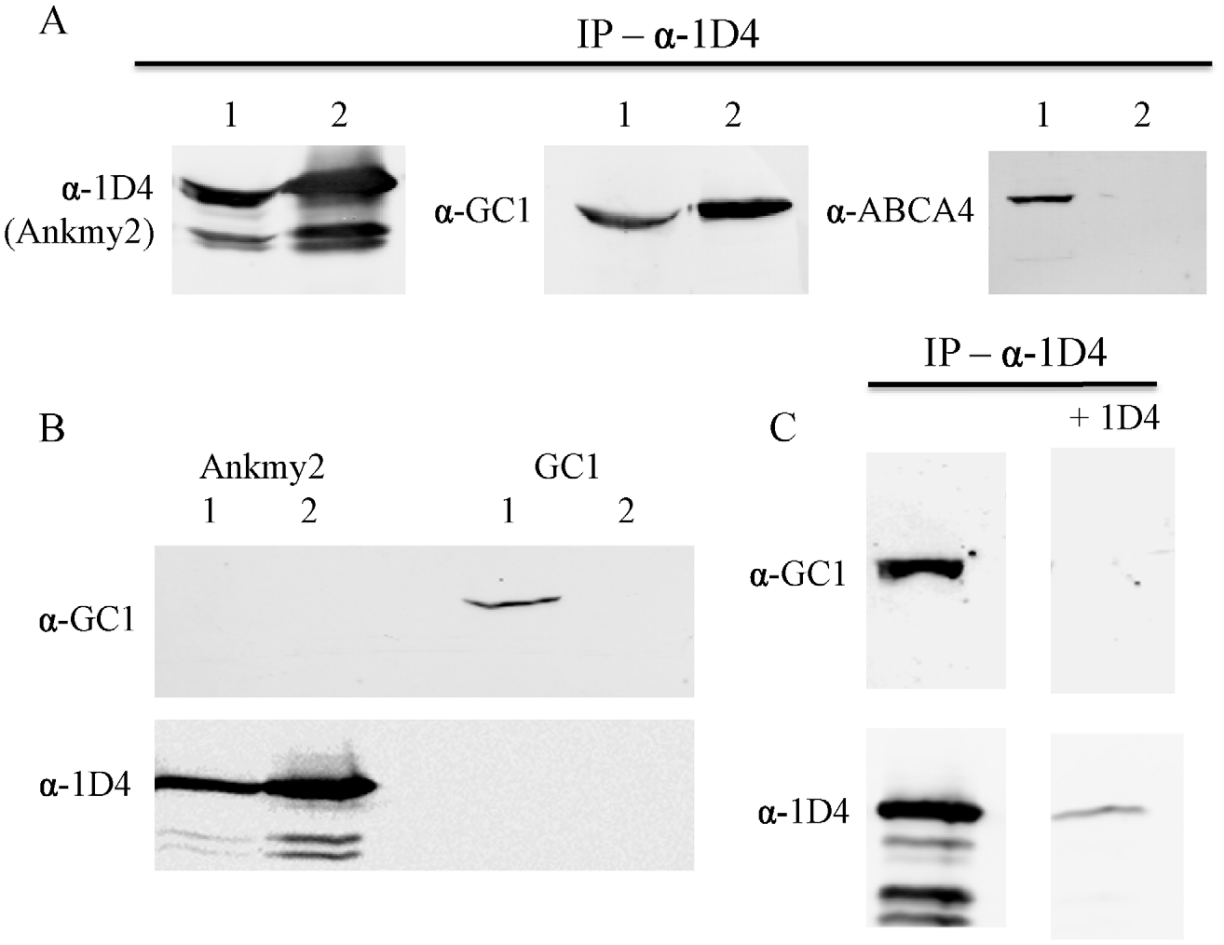
Ankmy2 and GC1 can be co-precipitated in HEK293 cells. In (A), detergent-solubilized extracts of HEK293 cells co-expressing Ankmy2-1D4 and either GC1 of ABCA4 were immununoprecipitated on a Rho 1D4-Sepharose matrix and the bound protein was analyzed on Western blots labeled with Rho 1D4 for detection of Ankmy2 and antibodies to GC1 or ABCA4 to detect co-precipitating proteins. Precipitation of 1D4-tagged Ankmy2 with 1D4 antibody also pulls down GC1 (retinal guanylyl cyclase), but not ABCA4 (retinal expressed ATP-binding Cassette, sub-family A, member 4). This indicates that GC1 forms a protein complex with Ankmy2, implying conservation of the functional interaction between DAF-11 and DAF-25. Lane 1 indicates the input proteins (whole cell lysate) and lane 2 indicates elution from immunoaffinity matrix. In (B) detergent-solubilized extracts of HEK293 cells expressing only Ankmy2-1D4 or GC1 were immunoprecipitated on a Rho 1D4 immunoaffinity matrix and analyzed on Western blots labeled with an anti-GC1 antibody or Rho 1D4 antibody. Lane 1: Input; lane 2: bound protein. The presence of Ankmy2-1D4 but not GC1 in the bound fractions indicates that GC1 does not nonspecifically interact with the Rho 1D4 immunoaffinity matrix. In (C), HEK293 cells co-expressing Ankmy2-1D4 and GC1 were co-immunoprecipitated on a Rho 1D4 immunoaffinity matrix in the absence or the presence of excess competing 1D4 peptide. Both Ankmy2-1D4 and GC1 bound in the absence of peptide. In the presence of the 1D4 peptide less than 10% of the Ankmy2 bound.

To ascertain if a functional association between DAF-25/Ankmy2 and guanylyl cyclase is evolutionarily conserved, we used a pull-down experiment to test whether mouse Ankmy2 interacts with the retinal-specific guanylyl cyclase GC1, a mammalian homolog of DAF-11 present within ciliary photoreceptors. We amplified Ankmy2 cDNA from a mouse retinal cDNA preparation (gift from Simon Kaja), and constructed a cDNA clone with the rhodopsin 1D4 epitope to use for co-IP experiments with anti-1D4 monoclonal antibody [40]. We co-expressed both in HEK293 cells to test for GC1 co-immunoprecipitation with the 1D4 epitope-tagged Ankmy2 (HEK293 cells do not express rhodopsin). Pull-down of Ankmy2 co-precipitated GC1, but not another control protein (retinal membrane protein ABCA4; Figure 6). This indicates that the functional interaction between DAF-25/Ankmy2 and guanylyl cyclase observed in ciliated sensory cells may be conserved between mouse and worm.

## Discussion

In this study, we have identified in a genetic screen for Dauer formation mutants a novel MYND domain-containing ciliary protein, DAF-25, that is required for the proper localization of a guanylyl cyclase (DAF-11) to sensory cilia. Disruption of DAF-25 does not interfere with intraflagellar transport (IFT) or ciliary ultrastructure, but the protein accumulates in a *che-11* retrograde IFT mutant. We therefore propose that DAF-25 is associated with IFT not as a ‘core’ protein but instead as an adaptor for transporting ciliary cargo. In our model, abrogation of DAF-25 would thereby not allow transport of DAF-11, which explains the improper localization of DAF-11 in *daf-25* mutants at the very base of cilia and the similarity in phenotype between *daf-11* and *daf-25* mutants.

The amino acid sequence and domain structure similarity between DAF-25 and Ankmy2 suggests an important function for the latter mammalian protein that may be similar to DAF-25 in *C. elegans*. We attempted to co-immunoprecipitate DAF-25 and DAF-11 in *C. elegans* but were unable to satisfactorily remove a sufficient amount of background proteins to avoid confounding any identified interaction (data not shown). We also showed that the retinal guanylyl cyclase GC1 binds to Ankmy2, and we propose that the functional relationship between DAF-25 and DAF-11 is conserved between Ankmy2 and GC1 in ciliated photoreceptor cells. Indeed, Ankmy2 may be required for the transport of not only GC1 but perhaps other cilia-targeted guanylyl cyclases as well as other cilia-targeted proteins in mammals. Further studies will be required to experimentally confirm whether Ankmy2 is required for transport of GC1 to the rod outer segment, and to test if Ankmy2 lesions result in retinal disease or a ciliopathy syndrome that includes retinopathies. Mutations in ciliogenesis and cilia related genes cause human disease phenotypes including Bardet-Biedl syndrome, retinopathies, obesity, *situs inversus* and polycystic kidney disease, among others [41], [42]. Interestingly, GC1 and the nuclear hormone receptor Nr2e3 shown to regulate Ankmy2 expression in mouse retina both harbor mutations in patients with retinal disease [43], [44].

While this research was being conducted we became aware of another group that cloned and characterized *chb-3* (Y48G1A.3/*daf-25*/Ankmy2) as a suppressor of the *che-2* body size phenotype [45]. Fujiwara *et al.*, (in press) describe the cloning of *chb-3/daf-25* and its essential role in GCY-12 cilia localization. They show that DAF-25 is required in a subset of sensory neurons to rescue the phenotypes they assayed (dauer formation and body size) using a *tax-4* promoter. This indicates that DAF-25 function is required in the neurons where cGMP signaling takes place (TAX-4 is a subunit of cGMP-gated calcium channel). They also show expression of DAF-25 in the ASJ neurons (one pair of neurons where DAF-11 is expressed) is required for rescue of the dauer phenotype, also indicating a cell autonomous role for DAF-25. It is interesting that screens for the Daf-c and Chb (*che-2* body size suppressor) phenotypes both resulted in the identification of *daf-25*/*chb-3* and separately identified its apparent ciliary cargos *daf-11* and *gcy-12*, guanylyl cyclases that specifically work in dauer formation and body size, respectively. This indicates that DAF-25/CHB-3/Ankmy2 may interact with cilia-targeted guanylyl cyclases in a general manner and that much of the phenotype of *daf-25*/*chb-3* mutants reflects a global defect in cGMP signaling, potentially along with other unidentified cargo proteins.

In conclusion, our findings uncover a novel ciliary protein that plays an important role in modulating the localization/function of cGMP signaling components, which are known to play a critical role in the function of ciliary photoreceptors [46]. DAF-25/Ankmy2 may also play a role in the ciliary targeting of other as of yet identified proteins. As such, Ankmy2 could participate in phototransduction and be associated with retinopathies, and more generally, could be implicated in other ciliary diseases (ciliopathies).

## Methods

### Mapping, Epistasis, and Phenotyping *daf-25*

*daf-25* mutations were created by treatment of N2 with 0.25 M EMS, or by *mut-2* transposon mobility, and selection for constitutive dauer formation as previously described [22]. For 3-factor mapping, *fog-1(e2121) unc-11(e47)* was crossed with *daf-25(m362)* and *daf-25(m362) unc-35(e259)* was crossed with *dpy-5(e61)*. Scoring the genotypes of the F2 progeny required the phenotyping of F3 progeny (due to the maternal effect of the *daf-25* dauer phenotype). Pooled SNP mapping was completed as previously described [30] with some changes. In the Po generation, CB4856 males were crossed to *daf-25;unc-11* double mutant hermaphrodites. The F1 males were crossed with CB4856 hermaphrodites. F2 hermaphrodites were selected by absence of Unc progeny. F3 hermaphrodites were placed one to a plate and were selected into wild type or mutant pools based on absence or presence of dauers in the F4. Wild type and mutant pools of F3 hermaphrodites were subject to SNP analysis as previously described [30]. ArrayCGH was done as previously described [47] for the leftmost 2.4 Mbp of Chromosome I with 50 base probes spaced every four base pairs.

Epistasis analysis was performed by crossing *daf-25(m362)* into *daf-12(m20)*, *daf-16(m26)*, *daf-3(mgDf90)*, *daf-10(e1387)* and *daf-6(e1377)*. Once the double mutants were isolated, the dauer phenotype was assayed to determine if *daf-25* was suppressed fully (no constitutive dauer larvae formed at 25°C), partially (fewer dauer larvae than *daf-25(m362)* control) or no suppression. Treatment with cGMP was performed as previously described [12] with 5 mM 8-bromo-cGMP (Sigma). Neuronal dye-filling was assayed by incubating a mixed-stage population of each genotype in Vibrant DiI (Molecular Probes) 1000-fold diluted in M9 buffer for one hour followed by washing in M9 and one hour destaining on plates. Chemotaxis assays were performed synchronized day-1 adults as previously described with the volatile attractants trimethyl-thiazole, pyrazine, benzaldehye and isoamyl alcohol [48].

The DAF-25::GFP construct was created by inserting the 2.0 kb promoter region 5′ of the AUG followed by *daf-25* cDNA the into the pPD95.77 vector (gift from Dr. Andrew Fire). After microinjection into N2 adults [49] with 10 ug/ml of pRF4 (contains *rol-6(su1006)*), and 90 ug/ml of DAF-25::GFP plasmid (described above), transgenics lines were established based on the roller phenotype. The extra-chromosomal array *mEX179*(p*daf-25::*DAF-25::GFP, *rol-6(su1006)*) was crossed into *daf-25(m362)* and rescue of the Daf-c phenotype was detected by normal non-dauer development in the F3 progeny grown at 25°C. GFP fluorescence was visualized on a Zeiss Axioskop with a Qimaging Retiga 2000R camera.

### Intraflagellar Transport and Ciliary Protein Localization Analyses

To measure the integrity of IFT within the *daf-25(m362)* mutant, kymograph analyses were performed using GFP-tagged CHE-11 and CHE-2 IFT markers. Time-lapse movies were obtained for the different strains, including N2, and kymographs were generated from the resulting stacked tiff images using Metamorph software (Universal Imaging, West Chester, PA). Rates of fluorescent IFT particle motility along middle and distal segments were measured as described previously [35], [50]. To assess how disrupting IFT affects the ciliary localization of DAF-25::GFP, *mEX179* was crossed into *che-11* mutants and visualized by microscopy essentially as described [35]. Fluorescence intensity was measured by analyzing images in ImageJ by highlighting the entire head region for each animal, then measuring pixel density minus the pixel density for an equal sized adjacent region. The localization of several GFP–tagged proteins in *daf-25(m362)* animals, namely DAF-11, OSM-9, TAX-4, CHE-2, CHE-11, CHE-13, BBS-8, OSM-5 and XBX-1, were ascertained by crossing the reporter into the mutant, followed by visualization using standard microscopy. Co-localization was carried out by injecting the *osm-5*p::XBX-1::tdTomato into *daf-25(m362)*;*daf-12(sa204)* and crossing it into TJ9386 which carries the DAF-11::GFP reporter [12].

### Electron Microscopy

Staged N2 and *daf-25* L2 larvae were produced by harvesting eggs from gravid adults by alkaline hypochlorite treatment, followed by overnight hatching in M9 buffer, and subsequent incubation of hatched L1 larvae on seeded NGM plates for 26 hours at 16°C. Worms were then washed directly into a primary fixative of 2.5% glutaraldehyde in 0.1 M Sorensen phosphate buffer. To facilitate rapid ingress of fixative, worms were cut in half using a razor blade under a dissecting microscope, transferred to 1.5 ml Ependorf tubes and fixed for one hour at room temperature. Samples were then centrifuged at 3,000 rpm for two minutes, the supernatant removed and the pellet washed for ten minutes in 0.1 M Sorensen phosphate buffer. The worms were then post-fixed in 1% osmium tetroxide in 0.1 M Sorensen phosphate buffer for one hour at room temperature. Following washing in Sorensen phosphate buffer, specimens were processed for electron microscopy by standard methods. Briefly, they were dehydrated in ascending grades of alcohol to 100%, infiltrated with Epon and placed in aluminum planchetes orientated in a longitudinal aspect and polymerized at 60°C for 24 hours.

Using a Leica UC6 ultramicrotome individual worms were sectioned in cross section from anterior tip, at 1 µm until the area of interest was located as judged by examining the sections stained with toluidine blue by light microscopy. Thereafter, serial ultra-thin sections of 80 nm were taken for electron microscopical examination. These were picked up onto 100 mesh copper grids and stained with uranyl acetate and lead citrate. Using a Tecnai Twin (FEI) electron microscope, sections were examined to locate, in the first instance, the most distal (anterior) region of the cilia, then to the more proximal regions of the ciliary apparatus. At each strategic point, distal segment, middle segment and transition zone/fiber regions were tilted using the Compustage of the Tecnai to ensure that the axonemal microtubules were imaged in an exact geometrical normalcy to the imaging system. All images were recorded, at an accelerating voltage (120 kV) and objective aperture of 10 µm, using a MegaView 3 digital recording system.

### Co-Expression and Co-Immunoprecipitation of Ankmy2 and Guanylate Cyclase 1 (GC1)

Mouse *ankmy2* cDNA, amplified from retinal RNA, was engineered to contain a sequence encoding a 9 amino acid 1D4 C-terminal epitope as previously described [40]. Ankmy2-1D4 and either human GC1 or the retinal ABC transporter ABCA4 as a control were co-expressed in HEK 293 cell. HEK 293 cell extracts were solubilized in 18 mM CHAPS in TBS (20 mM Tris, 150 mM NaCl, 1 mM EDTA, 1 mM MgCl_2_ and Complete inhibitor). The solution was stirred at 4°C for 20 minutes and subsequently centrifuged in an Optima TLA100.4 rotor (Beckman) for 10 minutes at 40,000 rpm to remove any residual unsolubilized material. The solubilized extract was applied to an immunoaffinity resin consisting of the Rho 1D4 antibody conjugated to Sepharose 2B [31]. After incubation at 4°C for one hour, the resin was extensively washed with TBS to remove unbound protein, and the bound proteins were eluted with 0.2 mg/ml of the 1D4 competing peptide in TBS for analysis by Western blot labeled with Rho 1D4 antibody for the detection Ankmy2-1D4 and antibodies to GC1 or ABCA4.

## Supporting Information

Figure S1 Lifespan phenotype of *daf-25*. Lifespan of *daf-25(m362)* does not significantly differ from wild type N2. Mean lifespan was 12.3 for *daf-25* (n = 96) compared to 13.2 for N2 (n = 88) while the maximum lifespan was 20 days for both (p = 0.08, t-test). Shown is one replicate of two. Survival was assayed at 25°C.
(1.56 MB TIF)

Figure S2 Dye filling of *daf-25* mutants. Dye filling assay showing *daf-25(m362)* and *daf-25(m98)* compared to the wild type N2. Worms were incubated for 1 hour in 0.1% DiI in M9 buffer. No difference was detected between the two *daf-25* alleles and the wild type N2.
(1.56 MB TIF)

Figure S3 Cilium ultrastructure is normal in *daf-25* mutants. Shown are TEM serial cross sections of an amphid channel from N2 and *daf-25(m362)* L2-staged worms. In the six pairs of images, low magnification images (B, D, F, H, J, L) are presented on the left and one axoneme from the left image is shown in high magnification on the right (C, E, G, I, K, M). (A) Schematic of an amphid pore and channel from wild-type adult N2 worms. 10 ciliary axonemes (only three shown in longitudinal section) extend from the distal dendrite tips (den) into the lumen of the amphid pore, which is created by channel cilia invaginating surrounding support cells (sheath, socket). Channel have a ∼1 µm long transition zone (tz) at the ciliary base, consisting of a constricted ring of 9 outer doublet microtubules (MTs), connected to the ciliary membrane via Y-link connections. This is followed by a ‘middle segment’ of ∼4 µm, consisting of a ring of 9 outer doublet MTs, along with a varying number of inner singlet MTs. At the middle segment tip, the B-tubule of each doublet MT terminates, with the A-tubule extending to form the characteristic singlet MT structure of the ‘distal segment’. (B–E) Distal segment region of amphid cilia showing that N2 (B, C) and *daf-25* (C–E) worms both possess 10 MT-singlet containing axonemes. (F–I) 4 µm (N2) or 5 µm (*daf-25*) proximal to B–E (through middle segments). Both N2 and *daf-25* animals possess axonemes of similar number and MT ultrastructure (e.g., doublet MTs). Interestingly, 9 outer doublet MTs are not always observed in N2 and *daf-25* worms (F, H), indicating that L2-staged worms lack a full complement of MTs (currently under investigation in Blacque lab). (J–M) 6 µm proximal to B–E (through transition zones and distal dendrites). Transition zones appear identical in N2 and *daf-25* worms, with Y-links (arrow) and the internal apical ring (arrowhead) clearly visible and intact. Scale bars; 200 nm.
(9.08 MB TIF)

Figure S4 Alignment of DAF-25 with Ankmy2. *C. elegans* (Ce) DAF-25 was aligned with *Homo sapiens* (Hs), *Bos taurus* (Bt), *Mus musculus* (Mm), and *Danio rerio* (Dr). The red bar indicates the ankyrin repeat domain and the blue bar indicates the zinc finger MYND domain. White font on black background indicates conservation in all five species, white font on grey indicates four, and black font on grey indicates three. Ankmy2 is very well conserved among chordates, with identity percentages compared to human Ankmy2 of 93% for cow, 88% for mouse, and 76% for zebrafish while DAF-25 shares 32% identity.
(2.18 MB TIF)

Figure S5 The *daf-25* transcript including allele and UTR information. Displayed is the sequence of the *daf-25* transcript including the molecular lesions in the four *daf-25* alleles. A line over the sequence indicates the extent of the deletion. A line under the amino acid sequence indicates the two protein domains including the ankyrin repeat domain in the first half of the sequence and the zinc-finger MYND domain near the C-terminus of the sequence.
(3.21 MB TIF)

Figure S6 Many cilia targeted proteins localize normally in *daf-25(m362)*. Shown are the localization patterns of the translational fusion constructs BBS-8::GFP, CHE-2::GFP, CHE-11::GFP, CHE-13::GFP, OSM-5::GFP and XBX-1::GFP. All six of these GFP-tagged proteins localize normally to the cilia in both N2 and *daf-25(m362)* mutants, indicating that DAF-25 is unlikely to be a core IFT complex component. For each genotype and transgenic construct the left panels are the anterior or amphid cilia and the right panels are the posterior or phasmid cilia. Arrowheads denote basal body regions whereas brackets show the ciliary axonemes.
(1.00 MB PNG)

Figure S7 DAF-11::GFP localization in N2, *daf-25(m362)*, and *daf-25(m362)*; *daf-12(sa204)*. Despite suppressing the dauer phenotype of *daf-25*, *daf-12* does not suppress the cilia mislocalization of DAF-11::GFP in *daf-25(m362)*. This indicates that entry into the dauer stage does not cause the mislocalization of DAF-11::GFP.
(0.88 MB TIF)

Table S1 Dauer Formation of *daf-25(m362)* compared to *daf-11(m84)*.
(0.03 MB DOC)
