# Supplementary figures and images for: Localization of a Guanylyl Cyclase to Chemosensory Cilia Requires the Novel Ciliary MYND Domain Protein DAF-25
Source: PLoS Genet. 2010 Nov 24;6(11):e1001199. doi: 10.1371/journal.pgen.1001199 (PMC2991253; doi:10.1371/journal.pgen.1001199)

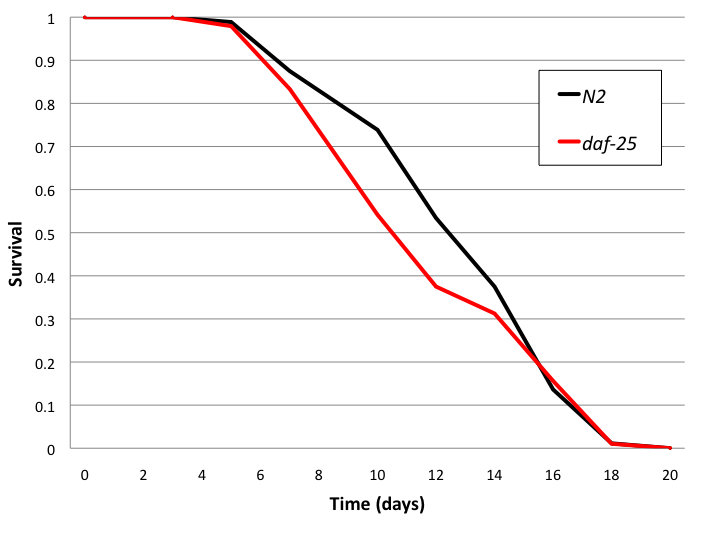

Supplement: Figure S1 — Lifespan phenotype of daf-25. Lifespan of daf-25(m362) does not significantly differ from wild type N2. Mean lifespan was 12.3 for daf-25 (n = 96) compared to 13.2 for N2 (n = 88) while the maximum lifespan was 20 days for both (p = 0.08, t-test). Shown is one replicate of two. Survival was assayed at 25°C. (1.56 MB TIF) [file pgen.1001199.s001.tif]

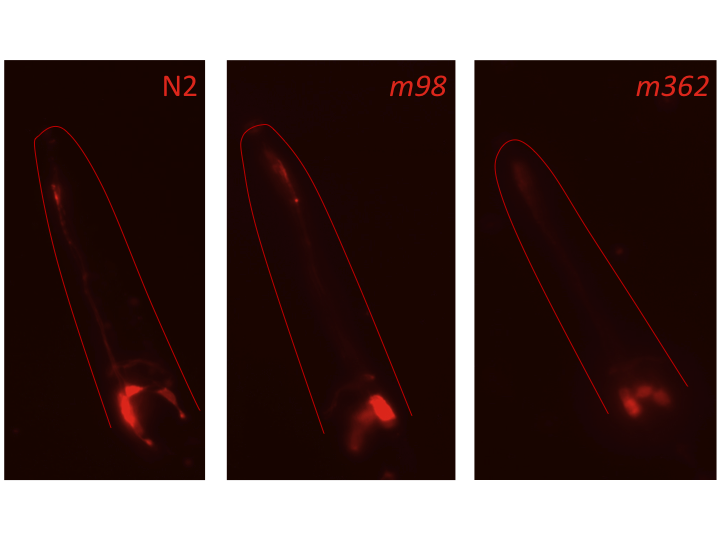

Supplement: Figure S2 — Dye filling of daf-25 mutants. Dye filling assay showing daf-25(m362) and daf-25(m98) compared to the wild type N2. Worms were incubated for 1 hour in 0.1% DiI in M9 buffer. No difference was detected between the two daf-25 alleles and the wild type N2. (1.56 MB TIF) [file pgen.1001199.s002.tif]

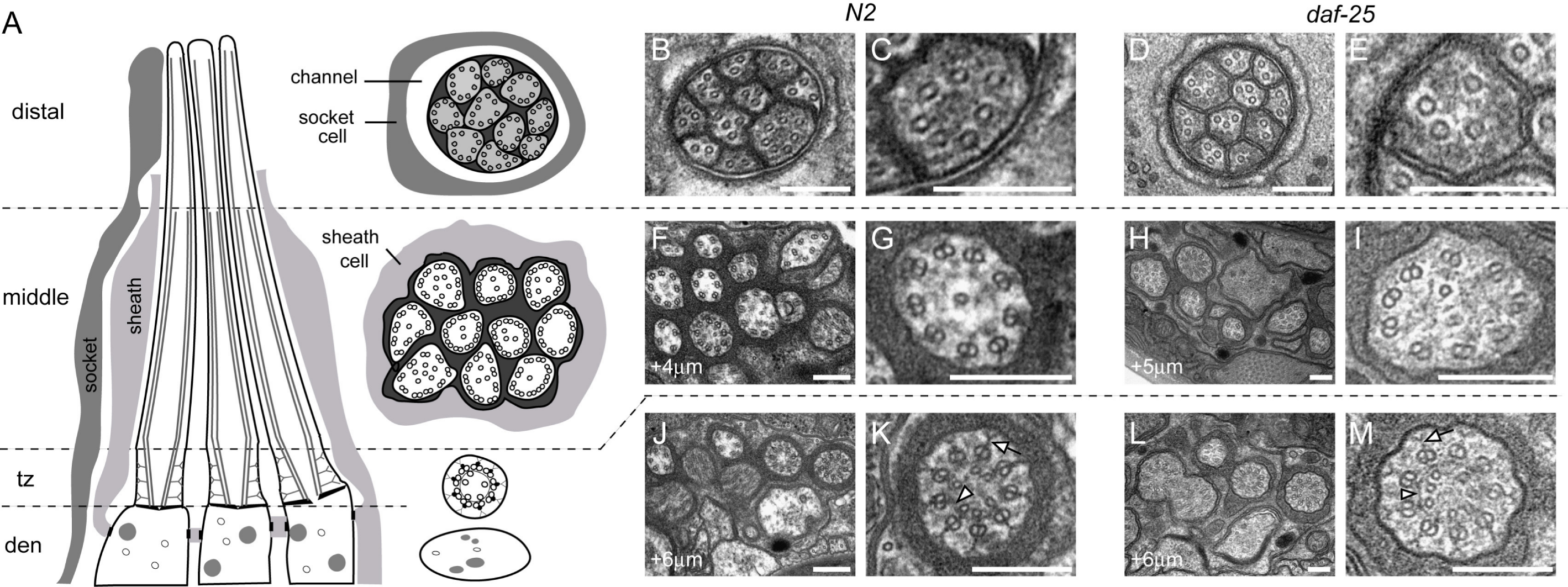

Supplement: Figure S3 — Cilium ultrastructure is normal in daf-25 mutants. Shown are TEM serial cross sections of an amphid channel from N2 and daf-25(m362) L2-staged worms. In the six pairs of images, low magnification images (B, D, F, H, J, L) are presented on the left and one axoneme from the left image is shown in high magnification on the right (C, E, G, I, K, M). (A) Schematic of an amphid pore and channel from wild-type adult N2 worms. 10 ciliary axonemes (only three shown in longitudinal section) extend from the distal dendrite tips (den) into the lumen of the amphid pore, which is created by channel cilia invaginating surrounding support cells (sheath, socket). Channel have a ∼1 µm long transition zone (tz) at the ciliary base, consisting of a constricted ring of 9 outer doublet microtubules (MTs), connected to the ciliary membrane via Y-link connections. This is followed by a ‘middle segment’ of ∼4 µm, consisting of a ring of 9 outer doublet MTs, along with a varying number of inner singlet MTs. At the middle segment tip, the B-tubule of each doublet MT terminates, with the A-tubule extending to form the characteristic singlet MT structure of the ‘distal segment’. (B–E) Distal segment region of amphid cilia showing that N2 (B, C) and daf-25 (C–E) worms both possess 10 MT-singlet containing axonemes. (F–I) 4 µm (N2) or 5 µm (daf-25) proximal to B–E (through middle segments). Both N2 and daf-25 animals possess axonemes of similar number and MT ultrastructure (e.g., doublet MTs). Interestingly, 9 outer doublet MTs are not always observed in N2 and daf-25 worms (F, H), indicating that L2-staged worms lack a full complement of MTs (currently under investigation in Blacque lab). (J–M) 6 µm proximal to B–E (through transition zones and distal dendrites). Transition zones appear identical in N2 and daf-25 worms, with Y-links (arrow) and the internal apical ring (arrowhead) clearly visible and intact. Scale bars; 200 nm. (9.08 MB TIF) [file pgen.1001199.s003.tif]

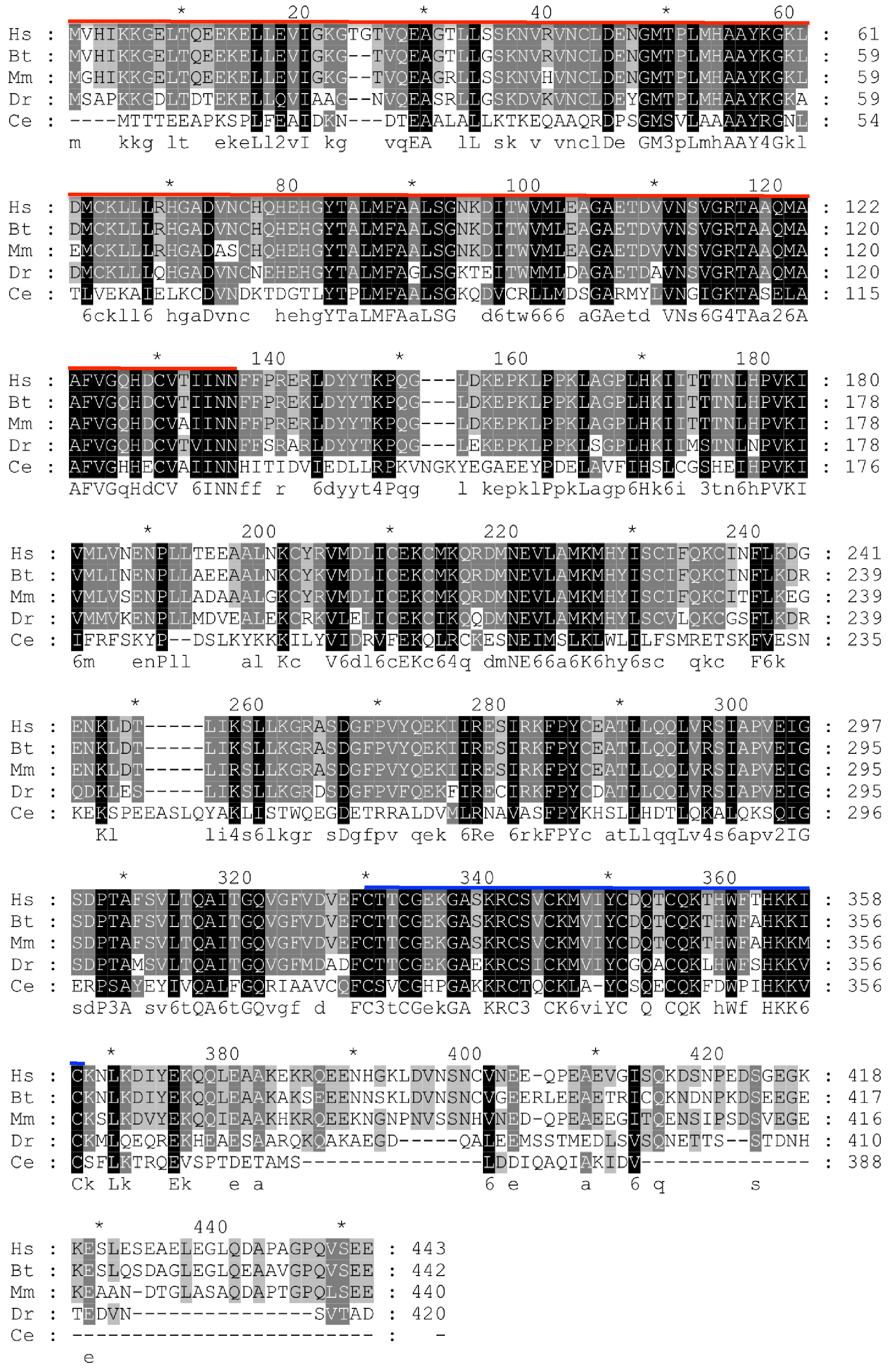

Supplement: Figure S4 — Alignment of DAF-25 with Ankmy2. C. elegans (Ce) DAF-25 was aligned with Homo sapiens (Hs), Bos taurus (Bt), Mus musculus (Mm), and Danio rerio (Dr). The red bar indicates the ankyrin repeat domain and the blue bar indicates the zinc finger MYND domain. White font on black background indicates conservation in all five species, white font on grey indicates four, and black font on grey indicates three. Ankmy2 is very well conserved among chordates, with identity percentages compared to human Ankmy2 of 93% for cow, 88% for mouse, and 76% for zebrafish while DAF-25 shares 32% identity. (2.18 MB TIF) [file pgen.1001199.s004.tif]

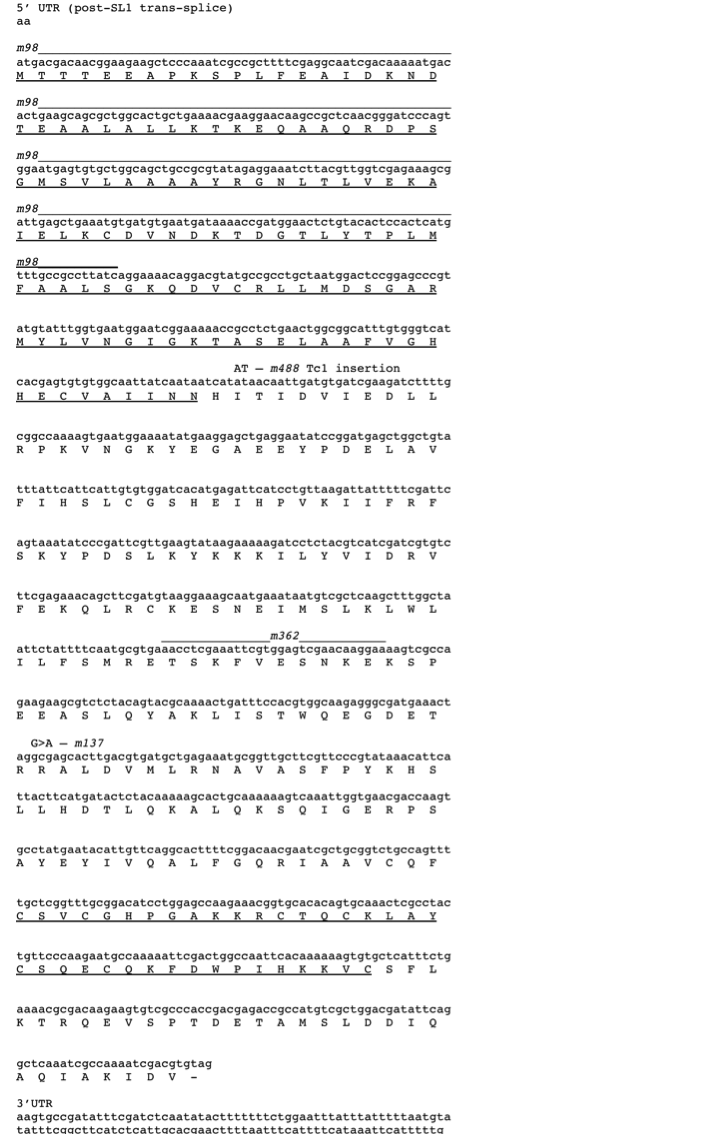

Supplement: Figure S5 — The daf-25 transcript including allele and UTR information. Displayed is the sequence of the daf-25 transcript including the molecular lesions in the four daf-25 alleles. A line over the sequence indicates the extent of the deletion. A line under the amino acid sequence indicates the two protein domains including the ankyrin repeat domain in the first half of the sequence and the zinc-finger MYND domain near the C-terminus of the sequence. (3.21 MB TIF) [file pgen.1001199.s005.tif]

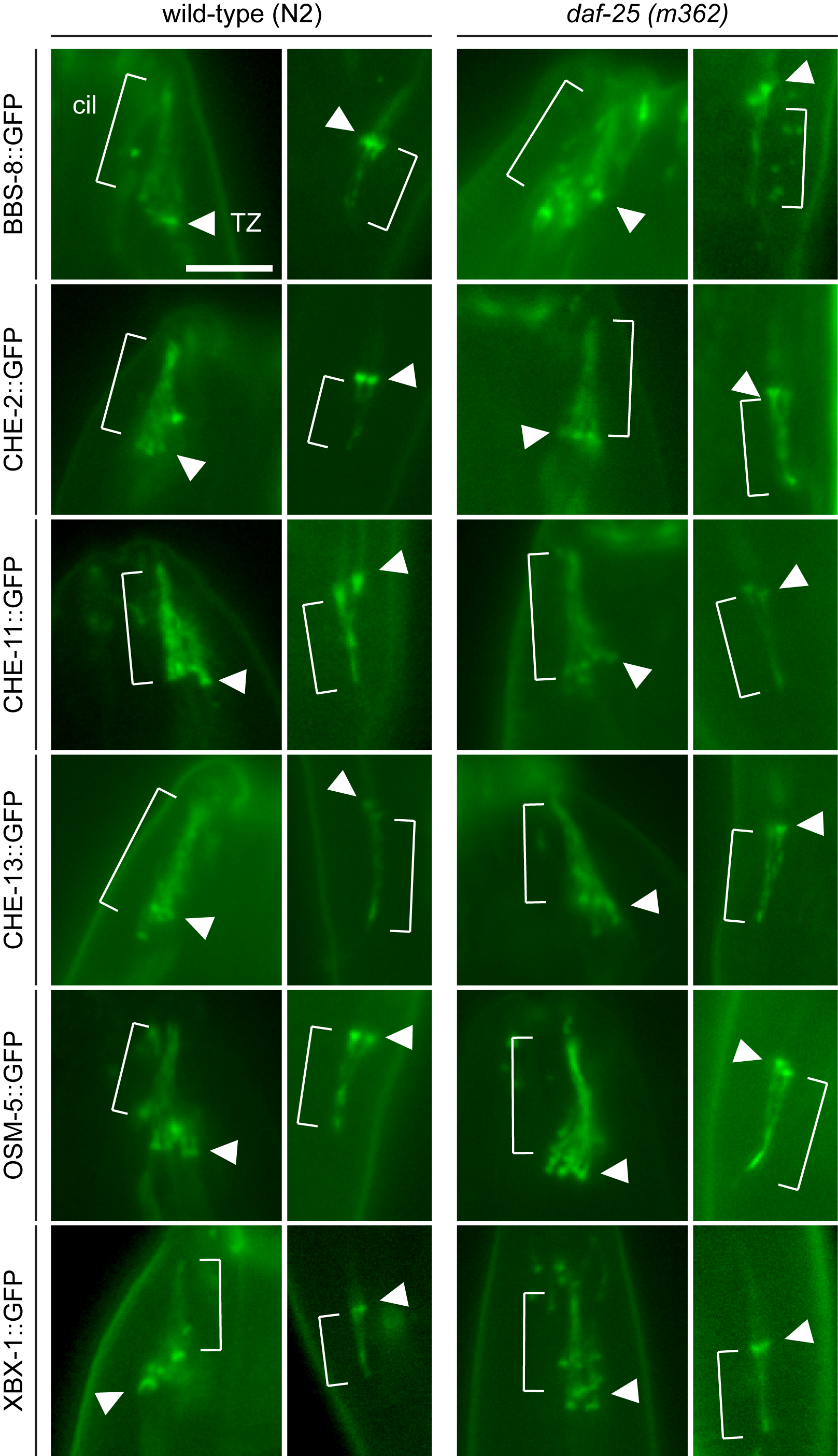

Supplement: Figure S6 — Many cilia targeted proteins localize normally in daf-25(m362). Shown are the localization patterns of the translational fusion constructs BBS-8::GFP, CHE-2::GFP, CHE-11::GFP, CHE-13::GFP, OSM-5::GFP and XBX-1::GFP. All six of these GFP-tagged proteins localize normally to the cilia in both N2 and daf-25(m362) mutants, indicating that DAF-25 is unlikely to be a core IFT complex component. For each genotype and transgenic construct the left panels are the anterior or amphid cilia and the right panels are the posterior or phasmid cilia. Arrowheads denote basal body regions whereas brackets show the ciliary axonemes. (1.00 MB PNG) [file pgen.1001199.s006.png]

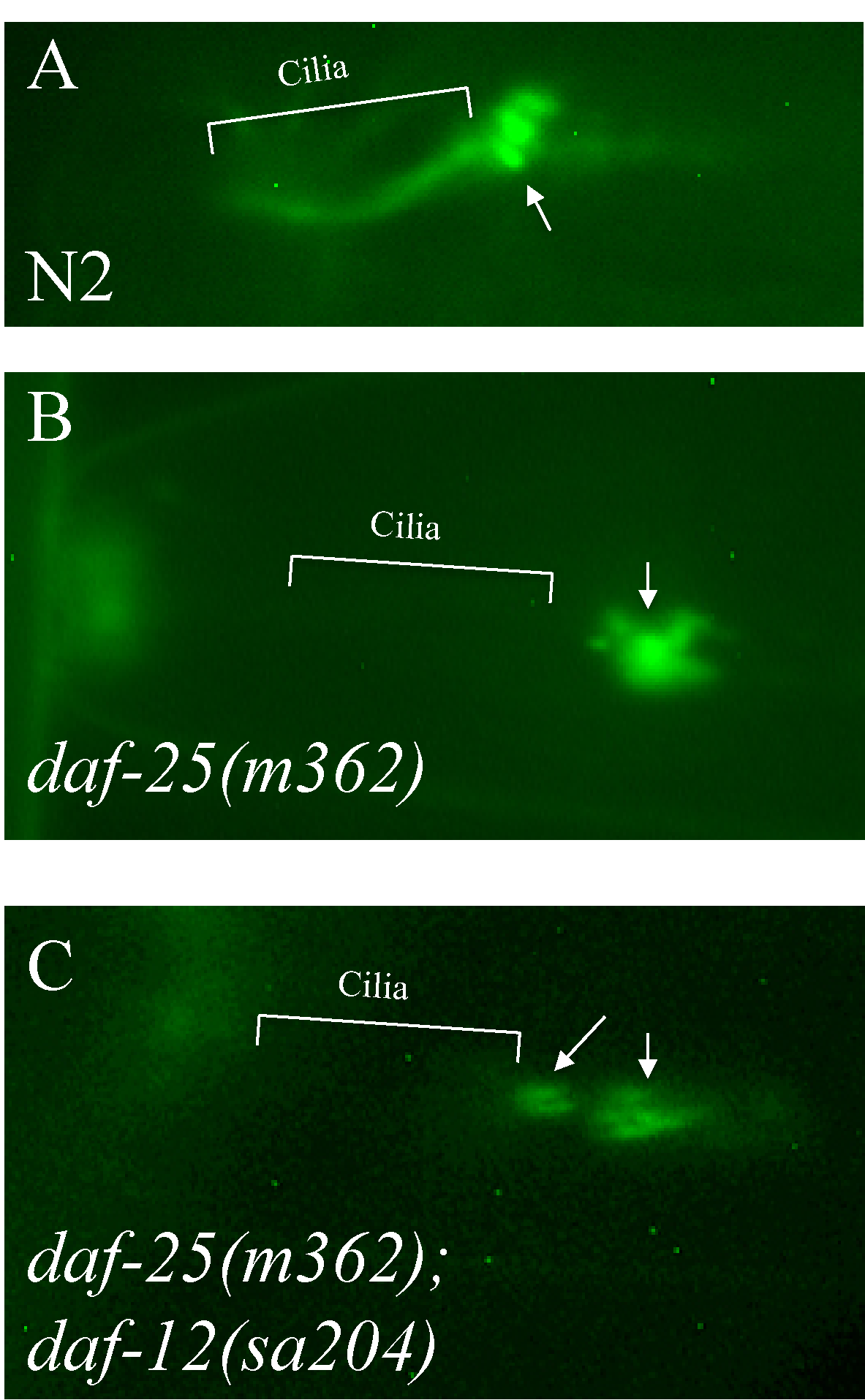

Supplement: Figure S7 — DAF-11::GFP localization in N2, daf-25(m362), and daf-25(m362); daf-12(sa204). Despite suppressing the dauer phenotype of daf-25, daf-12 does not suppress the cilia mislocalization of DAF-11::GFP in daf-25(m362). This indicates that entry into the dauer stage does not cause the mislocalization of DAF-11::GFP. (0.88 MB TIF) [file pgen.1001199.s007.tif]
