# Supplementary material for: Localization of a Guanylyl Cyclase to Chemosensory Cilia Requires the Novel Ciliary MYND Domain Protein DAF-25
Source: PLoS Genet. 2010 Nov 24;6(11):e1001199. doi: 10.1371/journal.pgen.1001199 (PMC2991253; doi:10.1371/journal.pgen.1001199)
Supplement: Table S1 — Dauer Formation of daf-25(m362) compared to daf-11(m84). (0.03 MB DOC) [file pgen.1001199.s008.doc]

| Genotype | % dauer 25 °C | N | % dauer 20 °C | N | % dauer 15 °C | N |
| --- | --- | --- | --- | --- | --- | --- |
| *daf-25(m362)* | 99.3% | 286 | 15.1% | 126 | 0% | >200 |
| *daf-11(m84)* | 99.1% | 343 | 15.6% | 77 | 0% | >200 |
